# Emerging Targets in Non-Small Cell Lung Cancer

**DOI:** 10.3390/ijms251810046

**Published:** 2024-09-18

**Authors:** Louisa Liu, Joshua Soler, Karen L. Reckamp, Kamya Sankar

**Affiliations:** 1 Samuel-Oschin Comprehensive Cancer Institute, Cedars-Sinai Medical Center, Los Angeles, CA 90048, USA; 2 Riverside School of Medicine, University of California, Riverside, CA 92521, USA

**Keywords:** non-small cell lung cancer, targeted therapies, oncogene driver, novel combinatorial approaches

## Abstract

Lung cancer is responsible for a high burden of disease globally. Over the last two decades, the discovery of targetable oncogenic genomic alterations has revolutionized the treatment landscape for early-stage and advanced non-small cell lung cancer (NSCLC). New molecular drivers continue to emerge as promising therapeutic targets, including KRAS non-G12C, RAF/MEK, HER3, Nectin-4, folate receptor alpha, ITGB6, and PRMT5. In this review, we summarize the emerging molecular targets with a potential clinical impact in advanced NSCLC, elaborating on their clinical characteristics and specific mechanisms and molecular pathways for which targeted treatments are currently available. Additionally, we present an aggregate of ongoing clinical trials investigating the available treatment options targeting such alterations, in addition to their current recruitment status and preliminary efficacy data. These advancements may guide further research endeavors and inform future treatment strategies to improve the management of and transform outcomes for patients with advanced NSCLC.

## 1. Introduction

**Figure 1:**
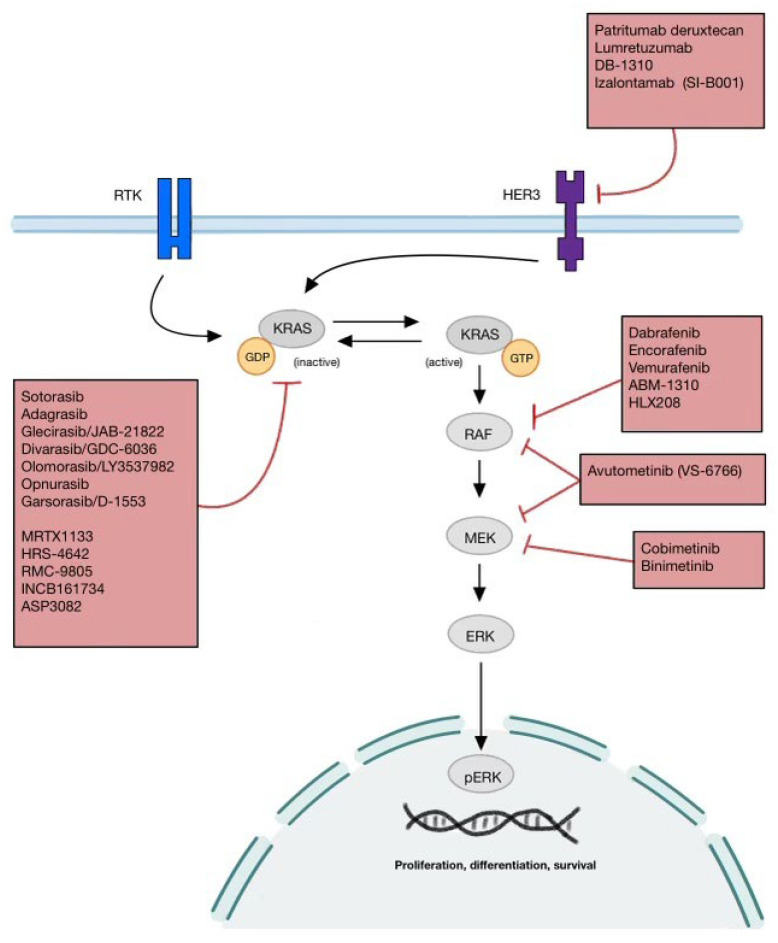
Depiction of the interplay of the major pathways to be discussed in this review (KRAS, BRAF/MEK, and HER3).

Lung cancer remains the leading cause of cancer-related death globally. The most common type of lung cancer is non-small cell lung cancer (NSCLC) with adenocarcinoma being the most common subtype. Lung cancer has been traditionally classified by histology and historically treated with chemotherapy as the mainstay of treatment. The identification of targetable oncogenic genomic alterations has significantly transformed the treatment paradigm for advanced or metastatic non-small cell lung cancer (NSCLC). Over the last two decades, several targets have been identified in lung adenocarcinoma (LUAD) including *EGFR* exon 19 deletion and L858R mutations, *ALK*, *ROS1*, and *NTRK* re-arrangements, *RET* rearrangements, *BRAF* V600E mutations, and *MET* exon 14 alterations, all of which have approved therapies available [1,2]. These targeted therapies have resulted in better outcomes and quality of life compared to chemotherapy in patients with tumors that harbor the oncogenic drivers. Recently, the spectrum of actionable genomic alterations (AGA) in LUAD has broadened to include *KRAS* G12C and *HER2* with the approval of new therapies [3,4,5,6]. Emerging molecular drivers continue to present promising therapeutic targets, including mutations in KRAS, RAF/MEK, HER3, Nectin-4, folate receptor alpha, ITGB6, and PRMT5 (Figure 1). This review aims to summarize the emerging therapeutic targets for NSCLC, elucidating their molecular pathways, therapeutic potentials, and ongoing trials.

## 2. KRAS

### 2.1. Molecular Pathway

*RAS* genes, including Kirsten Rat Sarcoma viral oncogene (*KRAS*), are proto-oncogenes encoding intracellular guanine nucleotide-binding proteins that belong to the GTPase family. RAS proteins are composed of two major domains: a catalytic domain called the G-domain, which binds guanine nucleotides and activates signaling, and a hypervariable region, which determines where the RAS proteins are localized on the cell membrane to perform their signaling function [7]. During signal transduction, RAS proteins act as molecular switches, cycling between on and off states. The guanosine triphosphate (GTP)-bound state is the active form while the guanosine diphosphate (GDP)-bound state is the inactive form. RAS-GTP binding acquires an altered conformation in the G-domain and activates a series of multiple signaling cascades [8,9,10]. Some of these pathways include RAF/MEK/ERK, PI3K/AKT/mTOR, RalA/B, and TIAM1/RAC1, which regulate cell proliferation, differentiation, and apoptosis. Approximately 90% of *KRAS* mutations are detected in exon 2. The most frequent mutations are the nucleotide substitutions in 12 codons of *KRAS* exon 2: p.G12C, p.G12D, and p.G12V [11]. These mutations impair the conversion from GTP-bound to GDP-bound state and lead to constitutive activation of downstream signaling independent of activation of upstream proteins [12]. *KRAS* may also play a role in modulating the tumor microenvironment to promote tumor progression as seen in preclinical studies in colorectal cancer. *KRAS* mutations have been associated with decreased major histocompatibility class I expression, upregulation of PD-L1, and promotion of an immunosuppressive immune cell population as myeloid-derived suppressor cells [13].

### 2.2. Clinical Characteristics

*KRAS* mutations are the most frequently detected gain-of-function mutations found in LUAD and have been historically considered “undruggable” [14]. *KRAS* mutations have been associated with a poor prognosis compared to *KRAS* wild-type tumors and carry an association with tobacco use [15]. The most frequent somatic mutation in NSCLC is *KRAS* G12C [16]. The KRAS G12C variant occurs more commonly in Western as opposed to Asian patients. Interestingly, there are significant differences in the distribution of KRAS somatic mutations by sex within the same ethnic groups, with the KRAS G12C variant occurring more frequently in White female patients than in White male patients and more frequently in Asian male patients than in Asian female patients with NSCLC [17].

### 2.3. Treatment

Due to the intrinsic characteristics of KRAS proteins including their lack of binding sites, small dimensions, and high affinity for GTP/GDP, KRAS remained a challenging therapeutic target for decades [18]. However, recent breakthroughs have led to the discovery of small molecules that bind covalently to mutant KRAS leading to subsequent formation of a new pocket in the switch-II region within the KRAS protein [19].

The identification of this allosteric switch II pocket has prompted the search for pharmacologic agents that target mutant forms of KRAS, particularly KRAS G12C. In the CodeBreak100 study, sotorasib showed an objective response rate (ORR) of 41% and progression-free survival (PFS) of 6.3 months in patients with advanced NSCLC [20]. In a phase III randomized trial of sotorasib versus docetaxel in previously treated patients with advanced NSCLC, sotorasib demonstrated increased PFS and a more favorable safety profile, however without a significant improvement in overall survival (OS) [21]. Similar results were reported with adagrasib from the phase 1 KRYSTAL-1 study where adagrasib demonstrated an ORR of 42.9% and PFS of 6.5 months [22]. The KRYSTAL-12 study randomized previously treated patients with advanced NSCLC to adagrasib and docetaxel, and similarly showed improvement in PFS and ORR with adagrasib [23]. The US Food and Drug Administration (FDA) granted accelerated approval for sotorasib in 2021 and adagrasib in 2022 for the treatment of previously treated patients with advanced NSCLC harboring *KRAS* G12C mutations based on these studies.

### 2.4. Novel Strategies to Target KRAS

*KRAS* G12C inhibitors: Newer *KRAS* G12C selective inhibitors intended to improve upon first-in-class inhibitors (sotorasib and adagrasib) are currently in clinical trials. Glecirasib (JAB-21822) exhibited potent anti-tumor efficacy both in vitro and in vivo as a single agent in preclinical models [24]. In a phase 2 single-arm trial in China evaluating 119 patients with locally advanced or metastatic *KRAS* G12C-mutated NSCLC who had progressed on platinum-based chemoimmunotherapy, glecirasib demonstrated an ORR of 47% and median PFS of 8.2 months (95% CI, 5.5–13.1) [25]. Glecirasib was well-tolerated with no dose-limiting toxicities (DLTs); the most common adverse effects were anemia, liver function abnormalities, and proteinuria. Divarasib (GDC-6036) demonstrated greater potency and selectivity for *KRAS G12C* in vitro compared to sotorasib and adagrasib in preclinical studies [26]. Divarasib demonstrated an ORR of 53.4% and PFS of 13.1 months in patients with NSCLC [27]. Treatment-related adverse events (TRAEs) occurred in 93% of patients, the majority of which (94%) were grade 1 or 2 severity. The most common TRAEs were nausea, diarrhea, and vomiting. Olomorasib (LY3537982) showed a range of anti-tumor activity from complete regression to significant tumor growth inhibition in xenograft models harboring *KRAS G12C* mutation [28]. Preliminary clinical data of olomorasib have reported an ORR of 60% and a favorable safety profile with absence of high-grade liver toxicity in patients with treatment-naïve *KRAS* G12C NSCLC [29] (NCT04956640) and have subsequently prompted the phase III trial SUNRAY-01 studying its combination with pembrolizumab with or without platinum-based chemotherapy based on PD-L1 expression level as the first-line treatment in patients with *KRAS* G12C-mutant advanced NSCLC (NCT06119581) [30]. Preclinical investigation of opnurasib (JDQ443) showed potent and selective anti-tumor activity in KRAS G12C cell lines and comparable efficacy to sotorasib [31]. Phase I data of opnurasib showed an ORR of 57% and DCR of 93% among 14 patients receiving the recommended dose of 200 mg twice daily (NCT04699188) [32]. A total of 71.4% of patients experienced a TRAE of any grade, with 7.1% of grade 3 and no grade 4–5 events at any dose level. The most common TRAEs were fatigue, edema, diarrhea, nausea, vomiting, and peripheral neuropathy. Opnurasib is currently being evaluated in a phase III randomized trial compared to docetaxel in previously treated patients with advanced NSCLC (NCT05132075) [33]. It is also being studied in a phase I/II trial in combination with the SHP2 inhibitor TNO155 or anti-PD1 antibody tislelizumab in patients with advanced *KRAS* G12C-mutated solid tumors (NCT04699188) [32]. Garsorasib (D-1553) demonstrated strong tumor growth inhibition with excellent central nervous system penetration preclinically [34]. Garsorasib demonstrated 40.5% ORR, 91.9% DCR, and 8.2 months median PFS in a phase I trial of patients in China with locally advanced or metastatic *KRAS* G12C-mutant NSCLC who were refractory to or intolerant of standard chemoimmunotherapy [35]. Across all doses of garsorasib, 95% of patients had TRAEs, most of which (57%) were grade 1 or 2 in severity. The most common TRAEs were liver function abnormalities, diarrhea, nausea, and vomiting. A total of 31% of patients required a dose interruption or reduction. Other *KRAS* G12C inhibitors such as ASP2453 are in preclinical development.

*KRAS* G12D inhibitors: The identification of small-molecule compounds that target KRAS-mutant proteins have inspired the rapid development of selective *KRAS* G12D inhibitors, which form salt bridges to bind to KRAS G12D in both its active and inactive states. The *KRAS* G12D inhibitor MRTX1133 has demonstrated potent in vitro and in vivo antitumor efficacy against *KRAS* G12D-mutant cancer cells in preclinical studies and is now being investigated in a phase I/II multiple expansion cohort trial in patients with advanced solid tumors harboring a *KRAS* G12D mutation (NCT05737706). Preclinical data have also suggested synergy with immunotherapy, as T-cells may augment the extent and duration of antitumor effect. The use of MRTX1133 in combination with checkpoint inhibitors targeting CTLA-4 or PD-1 resulted in enhanced tumor regression in xenograft models of pancreatic adenocarcinoma [36]. HRS-4642 exhibited anti-tumor efficacy in preclinical models as well as a disease control rate (DCR) of 94.4% in a phase I trial enrolling patients with advanced solid tumors harboring *KRAS* G12D mutations including 10 patients with NSCLC [37]. A third of the patients experienced a TRAE of grade 3 or higher, including hypercholesterolemia, increased lipase, and anemia. No treatment discontinuations or deaths due to TRAEs were reported. RMC-9805 (Revolution Medicines, Redwood City, CA, USA) and INCB161734 (Incyte) are other selective *KRAS* G12D inhibitors with promising preclinical results [38,39]. Their potential benefit as single agents or in combination with other anticancer therapies for patients with advanced or metastatic solid tumors with *KRAS* G12D mutation are under investigation in ongoing phase I trials (NCT06040541, NCT06179160).

*KRAS* degraders: Complementary therapeutic strategies aimed at targeting *KRAS* include *KRAS* degraders, which utilize cell degradation machinery to selectively eliminate mutant KRAS protein. Proteolysis-Targeting Chimeras (PROTACs) are heterobifunctional molecules that recruit a protein of interest, such as KRAS, to the cell’s ubiquitin-proteasome system for degradation [40]. ASP3082 (Astellas Pharma) is a PROTAC that binds to KRAS G12D and E3 ubiquitin ligase, leading to selective KRAS G12D protein degradation and growth inhibitory activity in *KRAS* G12D-mutated cancer cells while sparing *KRAS* wild-type cancer cells [41]. Its safety and tolerability is currently being studied in patients with NSCLC, colorectal cancer, pancreatic cancer, and other solid tumors with documented *KRAS* G12D mutations in a phase I trial [42].

Pan-RAS inhibitors: Pan-RAS inhibitors are designed to target multiple RAS isoforms at once. RMC-6236 (Revolution Medicines, NCT05379985) is a noncovalent inhibitor of the active GTP-bound state of several RAS variants. In vivo evaluations of RMC-6236 resulted in significant and durable tumor regression across multiple tumor types, particularly in NSCLC and pancreatic adenocarcinomas harboring *KRAS* glycine-12 substitutions (*KRAS* G12X) [43]. Preliminary phase I data of RMC-6236 demonstrated an ORR of 38% and DCR of 85% in 40 patients with previously treated advanced *KRAS* G12X-mutant NSCLC [44]. Across both NSCLC and pancreatic adenocarcinoma cohorts, the most common toxicities observed with RMC-6236 were rash, nausea, diarrhea, vomiting, stomatitis, and fatigue, nearly all of which were grade 1 or 2. QTX3034 (Quanta Therapeutics), RSC-1255 (RasCal Therapeutics), and YL-17231 (TEB-17231, Shanghai YingLi Pharmaceutical Co., Shanghai, China) are other pan-RAS inhibitors that have also demonstrated durable inhibition of *KRAS* signaling in vivo and tumor growth in vitro [45,46,47]. These agents are currently being studied in early-phase clinical trials of advanced solid tumors with *KRAS* mutations (NCT06227377, NCT04678648, NCT06078800) [48]. A comprehensive list of novel agents targeting *KRAS* is provided in Table 1.

Immunologic approaches: Other therapies targeting KRAS in development include neoantigen vaccines and adoptive cellular therapies. KRAS peptide vaccines are an emerging approach aimed at eliciting an immune response against cancer cells that harbor *KRAS* mutations. TG01 (Targovax), a peptide vaccine derived from KRAS-mutant proteins, has been tested in phase I trials of patients with multiple myeloma [49] and resected pancreatic cancer [50,51]. Efforts to expand the application of *KRAS* vaccines to NSCLC are ongoing. TG01 will be studied in a phase II trial in combination with daratumumab and nivolumab in patients with advanced refractory RAS-mutated NSCLC and pancreatic ductal cancer (NCT06015724). ELI-002 is a vaccine comprising lymph node-targeted amphiphile-modified G12D and G12R-mutant KRAS peptides together with a CpG oligonucleotide adjuvant. Preclinical data have shown increased cytotoxic T-cell responses and tumor clearance with ELI-002 [52,53]. The AMPLIFY-201 trial is a first-in-human phase I study of ELI-002 as treatment for patients with *KRAS*-mutated pancreatic ductal cancer and other solid tumors. Preliminary results from the trial included patients with *KRAS* G12D- or G12R-mutated pancreatic and colorectal cancer following surgery and chemotherapy, and demonstrated serum tumor biomarker reduction and notable immune responses in 80% of patients [54]. The safety and efficacy of ELI-002 will be further investigated in patients with high relapse risk *KRAS*-driven solid tumor types in the phase II AMPLIFY-7P trial (NCT05726864). Similarly, mRNA vaccines, which encode the mutant KRAS protein to induce an immune response, are being investigated as monotherapy or in combination with immune checkpoint inhibitors in *KRAS*-mutant advanced solid tumors including NSCLC (NCT03948763, NCT05202561) [55]. Other novel immunologic approaches like adoptive cellular therapies may offer patients a more personalized strategy for the management of NSCLC. These involve the generation of artificial T-cells or infusion of ex vivo expanded endogenous T-cells to improve therapeutic responses by enhancing tumor recognition and overcoming tumor-mediated immune suppression [56]. It remains to be seen whether the success of T-cell therapies that have revolutionized the treatment landscape of hematologic malignancies can be translated to solid tumors. The use of autologous engineered T-cells, tumor-infiltrating lymphocyte therapy, and T-cell receptor therapy have been introduced as potential treatments in NSCLC, though they remain in the early stages of development [56,57].

## 3. RAS/MEK

### 3.1. Molecular Pathway

*BRAF* mutations are diverse, with each mutation producing functionally distinct BRAF proteins. The altered BRAF proteins engage and activate the MAPK pathways in various ways. The initiation of the MAPK pathway will lead to RAS binding to GTP (RAS-GTP). Activated RAS triggers activation of the RAF family of kinases, which translocates to the cell membrane and forms homo- or heterodimers. Active RAF dimers autophosphorylate their activation loops and subsequently activate MEK dimers which in turn phosphorylate and activate ERK. Phosphorylated ERK then activates transcription factors which leads to cell growth, proliferation, differentiation, and survival. 

Mutations in the RAF kinase domains lead to constitutive activation of BRAF. A single-point mutation in the V600 position of the activation loop represents >90% of all cancer-related RAF mutations [58]. *BRAF* V600E mutation leads to constitutive RAF activation independent of RAS signaling and consequently increased tumor cell survival, proliferation, angiogenesis, and metastasis via the ERK signaling pathway. Importantly, with activation of phosphorylated ERK, negative feedback to upstream RAS and receptor tyrosine kinases does not allow for dimerization. Two regulatory elements of BRAF are the αC-helix and aspartate-phenylalanine-glycine (DFG) motif. Treatment with RAF inhibitors causes allosteric structural changes that lock these elements into specific conformations. 

### 3.2. Clinical Characteristics

Mutations in the mitogen-activated protein kinase (MAPK) pathway are implicated in the tumorigenesis of NSCLC. It is comprised of the kinases RAS, RAF, MEK, and ERK. Downstream receptors of the pathway, including RAF and MEK, have been studied as potential targets and offer promising therapeutic opportunities in NSCLC. *BRAF* mutations are identified in 6–8% of all cancers and in 1–2% of NSCLC [59]. The most common activating *BRAF* mutation is a point mutation in codon 600 (V600E) and is found almost exclusively in LUAD. There is no clear association of *BRAF* mutation with age, ethnicity, or sex [60]. Epidemiologic patterns are not clearly identifiable since *BRAF* mutation occurs in a small fraction of patients with advanced NSCLC [61]. Conflicting studies have reported smoking history to be or not be associated with *BRAF V600E* or non-*V600E* mutations [62]. Thus, all patients with advanced NSCLC, regardless of smoking history, should undergo broad molecular testing. *BRAF* mutation in NSCLC is often associated with increased programmed death ligand 1 (PD-L1) expression and tumor mutational burden (TMB) [63]. However, the prognostic implications of *BRAF* mutation in NSCLC are inconclusive. 

### 3.3. Treatments

Selective BRAF inhibitors: Vemurafenib, dabrafenib, and encorafenib were developed as highly selective BRAF inhibitors. They specifically bind to the active conformation of the BRAF kinase, occupying the ATP binding pocket and stabilizing the active conformation, leading to the potent inhibition of *BRAF* V600. Encorafenib exhibits activity against *BRAF* V600E, *BRAF* V600K, and wild-type *BRAF*. Dabrafenib and vemurafenib exhibit activity against *BRAF* V600E, V600K, V600R, and V600D mutations [61]. While BRAF-inhibitor monotherapy is effective initially, most patients develop resistance via reactivation of the MAPK pathway. It was theorized that the addition of MEK inhibition to BRAF inhibition could overcome the development of acquired resistance to BRAF inhibitors by blocking ERK signaling. BRAF inhibitors in combination with MEK inhibitors resulted in increased tolerability and efficacy, leading to several combination therapies being improved (dabrafenib plus trametinib, vemurafenib plus cobimetinib, and encorafenib plus binimetinib) in *BRAF* V600E-mutant melanoma [64,65,66].

In metastatic NSCLC, dabrafenib plus trametinib was approved as a first-line treatment for patients with BRAF V600E-mutant metastatic NSCLC based on a phase II study showing a median PFS of 10.2 months and OS of 18.2 months in previously treated patients and a median PFS of 10.8 months and OS of 17.3 months in treatment-naïve patients [67]. Recently, encorafenib plus binimetinib was also approved for BRAF V600E-mutant NSCLC based on the phase II PHAROS trial demonstrating an ORR of 75% in treatment-naïve patients and 46% in previously treated patients with metastatic NSCLC harboring a BRAF V600E mutation [68]. Vemurafenib has also shown significant efficacy in this setting in combination with cobimetinib as seen in the phase 2 basket study TAPUR [69,70], which reported an ORR of 42% and PFS 7.3 months, and the EURAF [71] trial, which reported an ORR of 53% and a median PFS of 5 months. The mechanisms of acquired resistance to BRAF inhibitors alone or in combination with MEK inhibitors are poorly understood but are thought to occur through bypassing or reactivation of the MAPK pathway or a parallel pathway such as the PI3K/AKT pathway [72].

### 3.4. Novel Strategies to Target BRAF/MEK

Pan-RAF inhibitors: Pan-RAF inhibitors, or type II inhibitors, stabilize the αC-helix of RAF proteins into the active “in” position and DFG motif into the “out” position [73,74]. They may overcome resistance mechanisms seen with single RAF inhibitors by preventing compensatory activation of other RAF isoforms. Notably, pan-RAF inhibitors may impose greater risk of side effects and toxicities given their broad pathway inhibition. Sorafenib, a multitargeted tyrosine kinase inhibitor (TKI), is the only first-generation type II RAF inhibitor approved for clinical use and is indicated for the treatment of advanced renal cell carcinoma, unresectable hepatocellular carcinoma, and metastatic differentiated thyroid carcinoma. Multiple trials over the years have been conducted to assess the efficacy of sorafenib in the treatment of patients with advanced NSCLC; however, the results across trials reveal inconsistent and questionable benefit in DCR, PFS, and OS [75,76,77]. Several other pan-RAF inhibitors, including belvarafenib [78], naporafenib [79] (LXH254), exarafenib [80], and LY3009120 [81] are being investigated in early phase trials to better define their efficacy and safety across various solid tumor types.

Novel mutation-specific BRAF inhibitors: Next-generation BRAF inhibitors target dimerization, which plays a significant role in the activation of wild-type and mutant BRAF and plays a role in the acquired resistance to BRAF inhibitors. Data from preclinical and clinical studies suggest that *BRAF* non-V600E mutations could be targeted more effectively with these inhibitors. ABM-1310, a water-soluble, cell-permeable novel small molecule BRAF inhibitor with high blood–brain barrier penetration, is currently being investigated in a phase I multi-center study for the treatment of BRAF V600-mutated solid tumors in the United States (NCT05501912) and in China (NCT04190628) [82]. Other ongoing drug development strategies include BDTX-4933 as monotherapy in advanced BRAF or *KRAS* non-G12C-mutant NSCLC (NCT05786924), PF-07799933 as monotherapy or in combination with binimetinib or cetuximab in BRAF-altered advanced solid tumors (NCT05355701), and the combination of vemurafenib plus the MEK1/2 inhibitor tunlametinib (H-085) in *BRAF*-altered advanced solid tumors (NCT03781219) [83,84]. Tovorafenib (TAK-580) is being studied in monotherapy or in combination with pimasertib (NCT01425008, NCT04985604) in recurrent, progressive, or refractory solid tumors with mutations in the MAPK pathway [85]. HLX208 is being studied as monotherapy in a phase II study of patients with *BRAF* V600-mutant advanced NSCLC in China (NCT05065398).

MEK inhibitors: MEK inhibition leads to the loss of negative feedback and promotes upstream RAS and RTK activation, subsequently restricting the efficacy of MEK inhibitor monotherapy and inducing treatment resistance. It has also been theorized that MEK1/2 inhibition may modulate the *KRAS*-mutant NSCLC tumor immune microenvironment via altered chemokine secretion and increased T-cell recruitment [86]. In addition to trametinib, binimetinib, and cobimetinib which have demonstrated enhanced anti-tumor activity and improved response rates in combination with their respective BRAF inhibitors in patients with metastatic *BRAF* V600E-mutant NSCLC, MEK inhibitors are currently being explored in combination strategies with other targeted or immunotherapeutic agents in NSCLC. For example, the addition of MEK to *KRAS* G12C inhibition, as seen with the combination of trametinib and sotorasib, has exhibited anti-tumor efficacy and an acceptable safety profile in advanced *KRAS* G12C-mutated solid tumors [87]. Specifically in the NSCLC cohort, treatment-naïve patients achieved a DCR of 86.7% (13/15), and patients treated with a prior KRAS G12C inhibitor achieved a DCR of 66.7% (2/3). The most common TRAEs across all patients included diarrhea, rash, nausea, and vomiting, which were predominantly grade 1 or 2. Discontinuation of sotorasib and/or trametinib due to a TRAE occurred in 24.4% of patients.

RAF/MEK clamps are small molecules that prevent the phosphorylation and subsequent activation of MEK by RAF kinases. Avutometinib (VS-6766) is a RAF/MEK clamp that has shown strong anti-tumor potency across tumor cell lines carrying various MAPK pathway alterations in preclinical studies. In *KRAS*-mutant NSCLC cell lines, avutometinib also demonstrated synergistic activity in reducing tumor cell viability when combined with other agents, including sotorasib, adagrasib, the focal adhesion kinase inhibitor defactinib, and the mTOR inhibitor everolimus. Although limited clinical activity was ultimately seen with its combination with defactinib in metastatic *KRAS* G12V NSCLC in the phase II RAMP202 trial [88], efforts to evaluate other avutometinib combinations in *KRAS*-mutant NSCLC are ongoing (NCT05074810, NCT05375994, NCT02407509). Preliminary data from the phase I/II RAMP203 trial demonstrated an ORR of 25% from the combination of avutometinib and sotorasib in *KRAS* G12C NSCLC (NCT05074810) [89]. Most TRAEs observed with this regimen were grades 1 and 2; the most common TRAEs of any grade included nausea, diarrhea, fatigue, and pruritus. A phase I trial studying the combination of avutometinib and everolimus has also shown encouraging preliminary efficacy and safety results, with an ORR of 20%, DFS of 90%, and median PFS of 6.35 months in advanced *KRAS*-mutated NSCLC [90]. At the recommended phase II doses, the majority of patients developed rash, creatine kinase elevation, mouth ulcers, or diarrhea of grade 1 or 2 severity. The most common grade 3 and 4 TRAEs were rash (18%) and pruritis (7%). A comprehensive list of currently enrolling clinical trials with novel RAF/MEK inhibitors is listed in Table 2.

Combination immunotherapy approaches: Studies have evaluated various combinations of anti-PD-1/PD-L1 therapies with BRAF and/or MEK inhibitors and have reported positive outcomes in solid tumors. In preclinical models, the use of MEK inhibitors reduced tumor cell proliferation, delayed tumor progression, and enhanced anti-tumor immunity of CD8^+^ T cells. Further activation of CD8^+^ T cells by synergistic targeting of PD-1/PD-L1 resulted in tumor regression and induced immunological memory [91,92]. The combination of cobimetinib plus atezolizumab across various solid tumors in a phase I/IB trial reported a median OS of 13.2 months and ORR of 18% in 28 patients with metastatic NSCLC [93]. Among all 150 patients enrolled in the study, the most common TRAE of any grade was diarrhea (67%), followed by rash (48%) and fatigue (40%). One patient died of sepsis, which was deemed to be related to treatment with atezolizumab. An active phase I trial is also investigating the combination of binimetinib and pembrolizumab in metastatic *KRAS*-mutant NSCLC [94]. A phase IB/II trial studying the combination of HLX208 with the anti-PD-1 antibody serplulimab (HLX10) in patients with *BRAF* V600E-mutant advanced solid tumors or NSCLC with PD-L1 TPS > 1% is also underway (NCT05641493). Another study is evaluating the combination of pembrolizumab with binimetinib in patients with metastatic or advanced NSCLC with PD-L1 > 50%, and initial results reported 36% of patients with partial response (NCT03991819) [95]. The most common toxicities were rash (82%), diarrhea (36%), and pruritis (36%). Some trials have investigated efficacy and safety of triple therapy combinations (BRAF/MEK inhibition in combination with anti-PD-1/PD-L1) in *BRAF*-mutant melanoma but have in general shown high rates of TRAEs [96,97].

## 4. HER3

### 4.1. Molecular Pathway

Unlike other family members, HER3 contains negligible kinase activity [98]. It possesses only a single ligand called heregulin or neuregulin. When a ligand binds to the extracellular region of HER3, this receptor interaction results in conformational changes facilitating dimerization, which subsequently activates downstream signaling pathways [99]. HER3 acts as a heterodimeric partner for other members of the EGFR family, namely EGFR and HER2 [100]. For example, HER3 becomes activated by dimerizing with mutated EGFR and mediates the PI3K/AKT pathway, which promotes cell survival, growth, and proliferation. The dimerization is often intensified during EGFR TKI treatment, which leads to therapy resistance and emphasizes the complexity of the EGFR/HER3 axis [101]. The most potent signaling occurs from the heterodimerization of HER3 with HER2, which functions to drive HER2-mediated PI3K downstream signaling. Increasing evidence supports HER3 to be an attractive target and inhibition of HER3 is now thought to be required to overcome therapy resistance and improve efficacy and outcomes in patients with metastatic NSCLC [102].

### 4.2. Clinical Characteristics

HER3, also known as ERBB3, is a member of the human epidermal receptor (HER) family of receptor tyrosine kinases, which also includes EGFR (ERBB1), HER2 (ERBB2), and HER4 (ERBB4) [103]. HER3 overexpression is observed in approximately 40% of NSCLC cases and is correlated with poor clinical outcomes [104,105]. HER3 activation leads to downstream PI3K/AKT pathway signaling, promoting cell survival and imparting resistance to EGFR-targeted therapies, which remains a cornerstone of treatment for EGFR-mutated NSCLC [106]. Activating mutations in HER3 have been identified and examined as direct therapeutic targets in NSCLC. While both EGFR and HER2 are excellent targets and a number of targeted therapies have been successfully transformed to the clinic for patients with *EGFR*- or *HER2*-mutated NSCLC, there is no Food and Drug Administration (FDA)-approved HER3-targeted therapy for cancer treatment.

### 4.3. Treatments

Treatment strategies are focused on the prevention of HER3 activation by inhibiting the kinase activity of its dimerization partner, impeding its dimerization with other ERBB members, or directly targeting the HER3 extracellular domain. The HER2-HER3 heterodimer is a potent oncogenic unit with remarkably high enzymatic potency and the ability to interact with multiple copies of downstream effectors [107]. As such, attention has been placed on targeting HER2-HER3 using TKIs against EGFR or HER2 such as lapatinib or anti-HER2 monoclonal antibodies like trastuzumab and pertuzumab. TKIs compete with ATP binding and prevent phosphorylation of the HER3 C-terminal tail. Trastuzumab and pertuzumab bind to the HER2 extracellular domain and prevent ligand-dependent dimerization between HER2 and HER3 [108]. However, many studies have implicated that these inhibitory effects are merely transient, and HER3 signaling resumes despite continued drug exposure and effective suppression of EGFR and HER2 due to AKT-mediated negative feedback signaling [109].

Given the upregulation of HER3 in resistance to EGFR- or HER2-targeted therapy, the use of HER3-targeted monotherapy or combination strategies has been suggested to overcome acquired resistance mechanisms and augment tumor inhibition [108,110]. Despite its lack of intrinsic kinase activity, HER3 can be targeted via direct blockade of its extracellular domain via antibodies. Patritumab was the first fully humanized anti-HER3 antibody and has shown promising results in early-phase clinical trials, including its ability to overcome heregulin-dependent resistance to EGFR TKIs in NSCLC [111]. Patritumab deruxtecan, an antibody–drug conjugate (ADC) which contains a topoisomerase I inhibitor payload, was investigated in the phase II HERTHENA-Lung01 study in patients with advanced EGFR-mutated NSCLC who had progressed following EGFR TKI and platinum-based chemotherapy [112]. Treatment with patritumab deruxtecan demonstrated clinical efficacy and durable intracranial responses, with an ORR of 29.8% (95% CI, 23.9–36.2), median PFS of 5.5 months, median OS of 11.9 months, and intracranial ORR of 33.3% (95% CI, 17.3–52.8). TRAEs of grade 3 or 4 occurred in 64.9% and 28.9% of patients, respectively. The most frequent grade ≥ 3 TRAEs were hematologic toxicities, including thrombocytopenia (20.9%) and neutropenia (19.1%). Treatment-related discontinuation due to adverse events was low (7.1%). A phase III randomized trial evaluating patritumab deruxtecan versus platinum-based chemotherapy after failure of EGFR TKI therapy is ongoing in metastatic or locally advanced EGFR-mutant NSCLC in the HERTHENA-Lung02 trial (NCT 05338970).

Lumretuzumab, another HER3-targeting antibody, was studied in a phase IB/II study in combination with carboplatin and paclitaxel as a first-line treatment in patients with advanced or metastatic squamous non-small cell lung cancer. In the three out of 12 patients who achieved a partial response, a higher expression of tumoral heregulin mRNA levels was reported [113]. All patients experienced at least one adverse event, with the most frequent TRAEs being diarrhea (75%), asthenia (66.7%), and neurotoxicity (41.7%). DB-1310 is another HER3 ADC comprising a novel humanized anti-HER3 immunoglobulin G1 (IgG1) monoclonal antibody, covalently linked to a proprietary DNA topoisomerase I inhibitor payload. DB-1310 demonstrated more potent anti-tumor activity than patritumab deruxtecan in vitro and in vivo [114]. Preclinical data support dual targeting of EGFR and HER3 to overcome acquired resistance to EGFR inhibition. An ongoing phase I trial is exploring patritumab in combination with osimertinib in advanced EGFR-mutated NSCLC [115]. Izalontamab (SI-B001) is a bispecific antibody which binds both EGFR and HER3 to inhibit the formation of EGFR × EGFR homodimers and EGFR × HER3 heterodimers and subsequently prevent the activation of downstream pathways. In a phase II study, SI-B001 demonstrated anti-tumor activity with manageable toxicity when combined with chemotherapy in locally advanced or metastatic EGFR/ALK wild-type NSCLC in the second-line setting [116]. Table 3 lists ongoing clinical trials evaluating *HER3*-targeted agents.

## 5. Nectin-4

### 5.1. Molecular Pathway

Nectin-4 is a cell adhesion molecule that belongs to the Nectin family, which also includes Nectin-1, -2, and -3 [117]. It comprises three parts: an extracellular region, a transmembrane region, and a cytoplasmic tail. The precise mechanism by which Nectin-4 participates in cancer progression is not clear. However, several studies have implicated the translocation of its intracellular domain into the nucleus via physical interaction with the nuclear transport receptor importin-a2, thus activating DNA repair and enhancing cell growth. In addition, the extracellular domain of Nectin-4 can increase angiogenesis via modulation of angiogenic markers. Other studies have confirmed its role in the adhesion, migration, and proliferation of tumor cells [118].

### 5.2. Clinical Characteristics

Nectin-4 plays a significant role in cancer development, mediating several functions including cell proliferation, differentiation, migration, and invasion [119]. Nectin-4 is highly expressed in various solid tumors and has emerged as a potential new biomarker and therapeutic target [120]. Nectin-4 is overexpressed in over 60% of NSCLC cases [121].

### 5.3. Treatments

Because of its high expression across multiple tumor types, Nectin-4 has emerged as an attractive target for the treatment of various cancers including NSCLC. Enfortumab vedotin, an ADC comprising a Nectin-4-directed antibody and microtubule inhibitor conjugate, first received approval for the treatment of patients with advanced or metastatic urothelial cancer. Its use is now being explored in other previously treated advanced solid tumors including NSCLC (NCT04225117) [122]. In a cohort of 43 patients with heavily pretreated NSCLC of non-squamous histology from the phase II EV-202 trial, enfortumab vedotin demonstrated an ORR of 14% and manageable safety profile with primarily grade 1 or 2 TRAEs (70%). The most common TRAEs were maculopapular rash (37%), peripheral neuropathy (37%), pruritus (35%), and alopecia (33%). Unfortunately, enrollment for those with squamous histology closed as only one of the 20 patients (ORR 4.3%) in the squamous cohort had achieved a response. BT8009 is a bicycle toxin conjugate consisting of a Nectin-4-targeting bicyclic peptide and cytotoxic payload that has demonstrated potent anti-tumor activity in rodent models [123]. The efficacy and safety of BT8009 in patients with Nectin-4-expressing advanced solid tumors will be studied in the Duravelo-1 study (NCT04561362). Table 4 lists currently-recruiting clinical trials with novel agents targeting Nectin-4, in addition to other targets including folate receptor alpha, ITGB6, and PRMT5.

## 6. Folate Receptor Alpha

### 6.1. Molecular Pathway

Folate receptor alpha (FRα) binds strongly to oxidized folate and, via potocytosis, transports it to the cytoplasm. Following folate uptake and internalization, FRα can translocate to the nucleus, where it can act as a transcription factor and directly regulate the expression of key development genes in cancer cells. This sets off a series of intracellular signaling cascades including the ERK and JAK/STAT3 pathway via phosphorylation and activates regulatory mechanisms involved in cell growth and proliferation. Furthermore, folate uptake can assist cancer cell proliferation and invasion by downregulating the expression of adhesion molecules such as E-cadherin, thus promoting cellular motility and metastasis.

### 6.2. Clinical Characteristics

FRα is a glycosylphosphatidylinositol (GPI)-anchored glycoprotein encoded by the gene *FOLR1*. It binds with high affinity to folic acid and its derivatives and mediates several cellular processes including cellular division, proliferation, and growth. High expression of FRα has been observed in several tumor types, including ovarian, endometrial, lung, and triple-negative breast cancer [124,125,126,127]. FRα was found to have a high discriminatory capacity based on the histologic subtype of lung cancer, with one study demonstrating an FRα positivity rate of 74% in adenocarcinomas vs. 13% in squamous cell carcinomas of the lung. Furthermore, high FRα expression was associated with improved overall survival in patients with LUAD who underwent surgical resection [128].

### 6.3. Treatments

Given its overexpression in NSCLC, FRα serves as a potential novel target for therapies using humanized monoclonal antibodies. Farletuzumab Ecteribulin (MORAb-202) is an ADC composed of a monoclonal antibody directed against FRα conjugated to the microtubule inhibitor eribulin. In a phase I study, Farletuzumab Ecteribulin was shown to have a DCR of 81.8% in patients with FRα-positive solid tumors (NCT03386942) [129]. TRAEs occurred in 95% of the patients, the most common of which were leukopenia (45%) and neutropenia (45%). Additionally, 23% of patients were identified to have grade 1 or 2 interstitial lung disease assessed to be related to MORAb-202. A phase II study investigating its use in patients with previously treated metastatic LUAD is actively recruiting (NCT05577715).

## 7. ITGB6

### 7.1. Molecular Pathway

Integrins are heterodimeric glycoprotein receptors that modulate intracellular signal transduction cascades in control of cell motility, survival, proliferation, and differentiation. They consist of alpha and beta subunits which span the cell membrane and have large extracellular domains, single transmembrane domains, and short intracellular tails. They mediate cell adhesion by binding to components of the extracellular matrix and transducing signals intracellularly. Activation of integrins results in conformational changes and triggers the formation of membrane extensions required for cell motility on ECM surfaces, migration of cells into sheets of other cells, and cell survival in foreign microenvironments [130,131].

*ITGB6* encodes the β6 subunit of integrin αvβ6, which works via its extracellular domain to convert latent transforming growth factor beta (TGF*β*) into its active form, which exerts growth inhibitory and transcriptional responses through the SMAD signaling pathway [132]. Deregulation of integrin αvβ6 and hereby TGF*β* signaling has been implicated in tumorigenesis and progression [133,134,135]. Moreover, TGF*β* is well known to promote T-cell suppression within tumor microenvironments and mediate resistance to immunotherapies [136,137].

### 7.2. Clinical Characteristics

The integrin-*β* superfamily (ITGBs) is comprised of eight members, *ITGB1–8*. *ITGB6* is overexpressed in numerous solid tumors of epithelial origin, including NSCLC, colorectal, gastric, and cervical cancers [138,139,140]. In these carcinomas, its expression has been correlated with increased disease progression and poor prognosis [138,141,142]. *ITGB6* is expressed at low levels in healthy adult lung epithelial tissue but is rapidly upregulated in areas of subclinical or clinical inflammation, suggesting its role in cell spreading, migration, and growth during morphogenetic events, tissue repair, and neoplasia [143].

### 7.3. Treatments

As a target for inducing an immune-mediated anti-tumor response, integrin αvβ6 has considerable therapeutic implications in NSCLC [138]. Sigvotatug Vedotin (SGN-B6A) is a novel ADC comprising a monoclonal antibody directed to integrin αvβ6 to deliver the cytotoxic payload monomethyl auristatin E (MMAE) to tumor cells. SGN-B6A induces immunogenic cell death, thus promoting the activation and recruitment of immune cells to the tumor environment [144]. Combinations with immune checkpoint inhibition may also represent a promising approach to harness the potential of integrin αvβ6 inhibition. An ongoing phase I trial is investigating the use of SGN-B6A alone and in combination with pembrolizumab, with or without chemotherapy, in patients with advanced solid tumors that are relapsed or refractory or intolerant to standard-of-care treatments (NCT04389632) [145]. A total of 113 patients with NSCLC treated with SGN-B6A monotherapy were included in the interim analysis, with a median of three prior lines of therapy. Preliminary efficacy results within the NSCLC cohort showed an ORR of 19.5% (95% CI, 12.6–28.0%), median DOR of 8.4 months (95% CI, 1.4–22.1), and median PFS of 3.5 months (95% CI, 2.7–4.9). Grade 3 or 4 TRAEs occurred in 46% of the patients, with the most common grade ≥ 3 TRAEs being dyspnea (9.7%), fatigue (7.1%), and neutropenia (5.3%). The promising single-agent activity of SGN-B6A seen has since prompted the initiation of the phase III SGNB6A-002 study, which will compare SGN-B6A to docetaxel in patients with previously treated advanced or metastatic non-squamous NSCLC (NCT06012435).

## 8. PRMT5

### 8.1. Molecular Pathway

The protein arginine methyltransferase (PRMT) family of enzymes is responsible for the methylation of arginine residues on histone proteins, a post-translational modification process that plays an important regulatory role in maintaining cellular homeostasis [146]. PRMT5 plays a complex role in oncogenesis, as its methylation of proteins is implicated in both tumor promotion and suppression across various tumor types [147]. In lung tumors, the PRMT5-driven arginine methylation of the N-terminal tails of histones H3 and H4 can modulate chromatin structure to induce transcriptional silencing of regulatory and tumor suppressor genes [148]. As seen in patient-derived xenograft models of LUAD, PRMT5 represses miR-99 family transcription via dimethylation of histone H4R3 [149,150]. This in turn increases FGFR3 expression, which activates its downstream molecules ERK1/2 and AKT to promote the growth, migration, and invasion of lung cancer cells [148,151].

Additionally, emerging evidence has shown the loss of tumor suppressor gene *CDKN2A* to be commonly accompanied by co-deletion of the proximal gene, *methylthioadenosine phosphorylase (MTAP)*, which encodes a major enzyme in the methionine salvage pathway [152]. *MTAP* deletion causes the accumulation of cellular metabolite methylthioadenosine (MTA), which binds to and partially inhibits PRMT5. As such, cancers harboring *MTAP* deletion are more sensitive to additional PRMT5 inhibition than normal cells [153].

### 8.2. Clinical Characteristics

The PRMT family is comprised of at least nine known members, PRMTs 1–9. PRMT5 is the primary enzyme responsible for the mono- and dimethylation of arginine and functions as a key player in a wide range of cellular and transcriptional processes including gene transcription, splicing, signal transduction, and cell cycle regulation [154]. PRMT5 overexpression has been implicated in tumor development and the progression of various cancers, including breast, hepatocellular, brain, and lung [155,156,157,158,159]. In NSCLC, growing evidence has also designated PRMT5 as a marker of poor clinical outcomes [155,160,161].

### 8.3. Treatments

PRMT5 has emerged as a synthetically lethal drug target for the treatment of *MTAP*-deleted cancers. The first generation of PRMT5 inhibitors that were developed did not demonstrate sufficient selectivity for MTAP-deleted cancer cell lines and resulted in intolerability from dose-limiting myelosuppression [162,163,164]. Next-generation PRMT5 inhibitors such as MRTX1719, TNG908, and TNG462 were subsequently designed to fully inhibit PRMT5 activity in MTAP-deficient cancer cells while sparing normal cells [165,166]. Following its encouraging preclinical data, MRTX1719 is now being explored in a phase I/II study in patients with advanced solid tumors with MTAP deletion (NCT05245500). Early results have confirmed ORRs in six of 18 patients (ORR 33.3%), including one patient with NSCLC harboring *EGFR* mutation and both *EGFR* and *MET* gene amplification who progressed after treatment with osimertinib and achieved a partial response after five cycles of MRTX1719 [165]. Similar phase I/II trials are currently investigating TNG908 and TNG462 for the treatment of advanced MTAP-deleted solid tumors (NCT05275478, NCT05732831) [166]. AMG193 is another second-generation PRMT5 inhibitor that selectively targets the MTA-bound state of PRMT5 that is enriched in MTAP-null tumors. It has demonstrated potent anti-tumor activity in MTAP-null cancer cell lines and tumor xenograft models and will be further studied in a first-in-human phase I/II trial in patients with advanced solid tumors with confirmed MTAP deletion (NCT05094336) [167].

## 9. Conclusions

Targeted therapy has successfully revolutionized the treatment paradigm of oncogene-driven early-stage and advanced NSCLC. Apart from the targets we have reviewed here, several others have been suggested including NSD3, ATR, FGFR, NRF2, AXL, and others [168]. Further, the evaluation of novel combinatorial strategies including targeted and immunologic treatment approaches is ongoing and will inform future treatment strategies. Treatment resistance and failure eventually occurs and remains the primary challenge for NSCLC-targeted therapeutics. The etiology of treatment resistance is complex and multifactorial and involves the complexity of intra- and inter-tumoral heterogeneity, mutations which develop from selective pressure of targeted therapy, and cancer genome evolution [169]. We expect the field of targeted therapy in NSCLC to continue to evolve, with a goal of optimizing personalized targeted approaches to further outcomes and survival in patients with metastatic NSCLC.

## Figures and Tables

**Table 1:** Novel KRAS inhibitors. Abbreviations: MOA (mechanism of action); NCT (National Clinical Trial number).

Drug	MOA	NCT	Phase	Study Design
Glecirasib/JAB-21822	KRAS G12C inhibitor	NCT05009329	II	Monotherapy
Divarasib/GDC-6036	KRAS G12C inhibitor	NCT04449874	I	Monotherapy
Olomorasib/LY3537982	KRAS G12C inhibitor	NCT04956640	I	Monotherapy
Olomorasib/LY3537982	KRAS G12C inhibitor	NCT06119581	III	With pembrolizumab +/− platinum-based chemotherapy, compared to placebo-control with pembrolizumab +/− platinum-based chemotherapy
Opnurasib	KRAS G12C inhibitor	NCT04699188	I/II	Monotherapy or in combination with tislelizumab
Opnurasib	KRAS G12C inhibitor	NCT05132075	III	Comparison with docetaxel
Garsorasib/D-1553	KRAS G12C inhibitor	NCT05383898	I	Monotherapy
MRTX1133	KRAS G12D inhibitor	NCT05737706	I/II	Monotherapy
HRS-4642	KRAS G12D inhibitor	NCT05533463	I	Monotherapy
RMC-9805	KRAS G12D inhibitor	NCT06040541	I	Monotherapy
INCB161734	KRAS G12D inhibitor	NCT06179160	I	Monotherapy or in combination with cetuximab or retifanlimab
ASP3082	PROTAC degrader	NCT05382559	I	Monotherapy or in combination with cetuximab or chemotherapy

**Table 2:** Novel agents targeting RAS/MEK pathway. Abbreviations: MOA (mechanism of action); NCT (National Clinical Trial number).

Drug	MOA	NCT	Phase	Study Design
RMC-6236	Pan-RAS inhibitor	NCT05379985	I	Monotherapy
ABM-1310	BRAF inhibitor	NCT04190628	I	Monotherapy and in combination with cobimetinib
ABM-1310	BRAF inhibitor	NCT05501912	I	Monotherapy
HLX208	BRAF V600E inhibitor	NCT05641493	IB/II	Combination with serplulimab
HLX208	BRAF V600E inhibitor	NCT05065398	II	Monotherapy
Avutometinib (VS-6766)	RAF/MEK clamp	NCT04620330	II	Monotherapy or in combination with defactinib
Avutometinib (VS-6766)	RAF/MEK clamp	NCT05074810	I/II	Combination with sotorasib
Avutometinib (VS-6766)	RAF/MEK clamp	NCT05375994	I/II	Combination with adagrasib
Avutometinib (VS-6766)	RAF/MEK clamp	NCT02407509	I	Monotherapy or in combination with everolimus
Cobimetinib	MEK inhibitor	NCT01988896	I/IB	Combination with atezolizumab
Binimetinib	MEK inhibitor	NCT03991819	I	Combination with pembrolizumab

**Table 3:** Novel agents targeting *HER3*. Abbreviations: MOA (mechanism of action); NCT (National Clinical Trial number).

Drug	MOA	NCT	Phase	Study Design
Patritumab deruxtecan	HER3 ADC	NCT04619004	II	Monotherapy
Patritumab deruxtecan	HER3 ADC	NCT 05338970	III	Comparison with platinum-based chemotherapy
Lumretuzumab	HER3-directed monoclonal antibody	NCT02204345	IB/II	Combination with platinum-based chemotherapy
DB-1310	HER3 ADC	NCT05785741	I/II	Monotherapy or in combination with trastuzumab
Patritumab deruxtecan	HER3 ADC	NCT04676477	II	Combination with osimertinib
Izalontamab (SI-B001)	Bi-specific HER3/EGFR monoclonal antibody	NCT05020457	II	Combination with platinum-based chemotherapy

**Table 4:** Novel agents targeting *Nectin-4*, *folate receptor alpha*, *ITGB6*, *and PRMT5*. Abbreviations: MOA (mechanism of action); NCT (National Clinical Trial number).

Drug	MOA	NCT	Phase	Study Design
Enfortumab vedontin	Nectin-4 inhibitor	NCT04225117	II	Monotherapy or in combination with pembrolizumab
BT8009-100	Nectin-4 inhibitor	NCT04561362	I/II	Monotherapy or in combination with pembrolizumab
Farletuzumab ecteribulin (MORAb-202)	Folate receptor alpha ADC	NCT03386942	I	Monotherapy
Farletuzumab ecteribulin (MORAb-202)	Folate receptor alpha ADC	NCT05577715	II	Monotherapy
Sigvotatug Vedotin (SGN-B6A)	ITGB6 inhibitor	NCT04389632	I	Monotherapy or in combination with pembrolizumab +/− platinum-based chemotherapy
Sigvotatug Vedotin (SGN-B6A)	ITGB6 inhibitor	NCT06012435	III	Combination with docetaxel
MTRX1719	ITGB6 inhibitor	NCT05245500	I/II	Monotherapy
TNG908	PRMT5 inhibitor	NCT05275478	I/II	Monotherapy
TNG462	PRMT5 inhibitor	NCT05732831	I/II	Monotherapy
AMG193	PRMT5 inhibitor	NCT05094336	I/II	Monotherapy
